# Cognitive performance, creativity and stress levels of neurotypical young adults under different white noise levels

**DOI:** 10.1038/s41598-022-18862-w

**Published:** 2022-08-26

**Authors:** Mohamad Awada, Burcin Becerik-Gerber, Gale Lucas, Shawn Roll

**Affiliations:** 1 Department of Civil and Environmental Engineering, University of Southern California, Los Angeles, CA 90089 USA; 2 USC Institute for Creative Technologies, University of Southern California, Los Angeles, CA 90089 USA; 3 Chan Division of Occupational Science and Occupational Therapy, University of Southern California, Los Angeles, CA 90089 USA

**Keywords:** Civil engineering, Human behaviour, Occupational health

## Abstract

Noise is often considered a distractor; however recent studies suggest that sub-attentive individuals or individuals diagnosed with attention deficit hyperactivity disorder can benefit from white noise to enhance their cognitive performance. Research regarding the effect of white noise on neurotypical adults presents mixed results, thus the implications of white noise on the neurotypical population remain unclear. Thus, this study investigates the effect of 2 white noise conditions, white noise level at 45 dB and white noise level at 65 dB, on the cognitive performance, creativity, and stress levels of neurotypical young adults in a private office space. These conditions are compared to a baseline condition where participants are exposed to the office ambient noise. Our findings showed that the white noise level at 45 dB resulted in better cognitive performance in terms of sustained attention, accuracy, and speed of performance as well as enhanced creativity and lower stress levels. On the other hand, the 65 dB white noise condition led to improved working memory but higher stress levels, which leads to the conclusion that different tasks might require different noise levels for optimal performance. These results lay the foundation for the integration of white noise into office workspaces as a tool to enhance office workers’ performance.

## Introduction

Brain signals are characterized by multitudes of noisy neural inputs and outputs. The role of the central nervous system is to differentiate between the information-carrying component in the neural signal and irrelevant noise that interferes with that signal. Still, the irrelevant noise remains a vital element for successful intraneuronal communication, as the central nervous system uses it to increase the signal-to-noise ratio^1^ (i.e., signal power to noise power) which enhances the internal neural signals^2^. The Moderate Brain Arousal (MBA) postulates that moderate levels of external auditory white noise introduce internal noise to the neural systems which allow undetected neural signals to pass the detection threshold thus leading to better cognitive performance^3,4^. Hence, the MBA model hypothesizes that the internal neural noise and external white noise work additively based on the stochastic resonance concept. Stochastic Resonance (SR) is a phenomenon in which signal processing is improved by adding random noise (e.g., white noise). In other words, signals that are below the threshold of detection become detectable when a random (stochastic) noise is added^5^. The MBA model also suggests that dopamine levels modulate this neural noise^6^. Low dopamine levels cause dampening in neural responses that decrease the overall neural noise and lead to failure in sustaining attention and as such weakening cognitive performance^7^. Thus, to optimize cognitive functioning, stochastic resonance requires high white noise levels when internal neural noise is low (low dopamine levels) but demands less white noise when dopamine activity is high^3,4,8–10^.

Such findings made researchers recognize the importance of using white noise to improve the attention span of individuals with Attention Deficit Hyperactivity Disorder (ADHD), who are generally associated with low dopamine levels (weak internal neural noise). Thus, the MBA model suggests that people diagnosed with ADHD demand more white noise than neurotypical individuals for SR to take place^11^. For instance, children with ADHD exposed to white noise (80 dB) showed better memory recall capacities^12^. Additionally, Helps et al.^13^ concluded that participants rated by their teachers as sub-attentive performed better with the working memory test and Go-No-Go task when they were subject to white noise (75 dB and 85 dB). Despite its potential, most studies investigating the effect of white noise on cognitive performance have focused on children diagnosed with ADHD, while a limited number of studies, as elaborated below, have investigated the effect of exposure to white noise on the cognitive performance of neurotypical adults. Although the MBA model suggests that healthy individuals with proper internal neural noise do not necessarily require white noise to optimize their cognitive performance^6^, recent works suggest otherwise. For example, a study conducted on neurotypical individuals showed that those who are frequently exposed to white noise present improved cognitive performance^3^. Furthermore, Othman et al.^4^ concluded that low intensity white noise enhanced the auditory working memory performance of 20 healthy adults. Finally, Angwin et al.^9^ found that participants who were exposed to white noise showed higher attentional capacity, enhanced lexical acquisition and improved recall accuracy compared to a non-noise condition.

Given the potential of white noise in boosting cognitive functions, the primary aim of this study is to investigate the effect of different white noise levels on the cognitive performance of neurotypical adults in office spaces. We focus on workers associated with office and administrative work as they represent a major part of the general workforce around the world and specifically in the U.S^14^. Office work is often associated with knowledge-based work^15^. Such work flourishes in workspace environments that promote optimal conditions for better cognitive capabilities (e.g., learning, thinking, reasoning, remembering, attention, perception and executive function^16–18^) Additionally, cognitive skills are a necessity to overcome work-related challenges and create high-quality work.

Moreover, employees’ creativity has always been the most important asset for the success of businesses, as it is essential for organizational growth and development^19^. Hillier et al.^20^ suggest that high level of white noise leads to increased stress levels which results in deteriorated creativity. Similarly, Martindale and Greenough^21^ concluded that a 75 dB white noise can cause a decrease in creative thinking among people. However, Toplyn and Maguire^22^ found contradicting results. After assessing their creativity levels using the Remote Associate Test (RAT), participants were asked to complete other creativity-related tasks under three conditions: low, moderate, and high white noise. Their results suggest that highly creative participants, scoring high on the RAT test, demonstrated the highest creative tendencies when exposed to moderate white noise levels. In summary, there have been a few studies that investigated how white noise influences creativity with mixed results. In fact, in their review, Mehta et al.^23^ concluded that white noise is generally associated with reduced creativity levels, but pointed out that more research is needed. Thus, a secondary aim of this study is to determine how different white noise levels affect the performance of neurotypical office workers’ creativity.

Finally, studies have shown that exposure to high levels of white noise can lead to stressful reactions manifested by physiological arousal. In their study, Nakajima et al.^24^ used white noise (70 dB) as an auditory-stress inducing mechanism while studying how music can help individuals recover from a stressful situation. Their results suggest that white noise was associated with an increase in heart rate. Stress responses have also been demonstrated in other studies when participants were introduced to white noise between 75 and 93dB^25^ and at 90 dB^26^. In the latter study, when exposed to 90 dB for 15 min participants demonstrated a significant increase in the secretion of salivary chromogranin A, a protein used as an indicator of stress. Most of the related literature has focused on relatively high levels of white noise, which is why a tertiary aim of this study is to determine the effect of different white noise levels on the physiological responses and stress levels of neurotypical office workers.

In this study, we assess the effect of two white noise conditions, white noise at 45 dB and white noise level at 65 dB, on the cognitive performance, creativity, and physiological responses of neurotypical adults in a private office space. Among the different cognitive functions, we focus on sustained attention, selective attention, inhibition, working memory as well as speed and accuracy in completing tasks as these functions are crucial to the success of the daily office tasks. Electrodermal activity (EDA) is used to assess participants’ physiological responses. The two-white noise conditions are compared to a baseline condition where participants are exposed to ambient noise in the office. The paper is organized as follows: “Methodology”section outlines our methodology; “Results”section introduces the results; “Discussion”section provides discussions and analysis. Finally, “Conclusions”section is a summary of our conclusions.

## Methodology

### Participants

Forty adults participated in this study voluntarily. A power analysis was conducted using G*Power version 3.1.9.7 to determine the sample size^27^. For an effect size (*f* = 0.2) and an *α* error probability = 0.05, the obtained sample was sufficient for a power of 80%. Of the 40 participants, 24 identified themselves as male and 16 as female. The average age of the participants was 25.82 ± 7.53. Also, 37 specified that they consider their right hand as the dominant one, and the remaining 3 indicated that their left hand is dominant. All 40 participants were graduate students at the University of Southern California. The study was limited to participants between 18 and 64 years old. Individuals with visual problems, hearing deficits, noise sensitivity, and/or physical injuries, making it uncomfortable to sit for a long period, were not eligible to participate. A screening survey was used for determining eligibility. If a participant felt uncomfortable during the experiment, they were given the option to discontinue at any point. Every participant performed an online hearing screening^28^ to ensure they had normal hearing sensitivity in both ears. This test consists of three main parts: (1) four self-evaluation questions about hearing abilities, (2) tone testing at 500, 1000, and 4000 Hz, and (3) conversation comprehension where participants would listen to a short conversation and respond to related questions^28^. At the end of the test, participants would be provided with a hearing report stating their hearing loss level. Only participants with no hearing loss were eligible to be part of the experiment. One participant was excluded from the analysis due to unrealistically fast response speed on survey responses; data from the remaining 39 participants were included in all analyses. The study was approved by the Institutional Review Board of the University of Southern California (UP-20-00389 IRB study number). All participants reviewed the informed consent and agreed to participate in the experiment. All experiments were performed in accordance with relevant guidelines and regulations.

### Auditory conditions

The experiment consisted of three auditory conditions: white noise at 45 dB, white noise at 65 dB, and ambient noise. According to the Center for Disease Control and Prevention (CDC), people exposed to a 70 dB noise level for a prolonged time might feel overwhelmed and annoyed^29^. To eliminate this effect on our experimental results, we chose a 65 dB as the high white noise condition. On the other hand, the U.S. Environmental Protection Agency (EPA) recommends a 45 dB for indoor activities which is why a 45 dB was chosen our low white noise condition^30^. White noise was presented via Bose QuietComfort 35 headphones with active noise cancellation to reduce background noise. The white noise was generated using Audacity software version 3.0.2; this software has been used previously by several research studies to generate white noise tracks^4,8,9^. The experiments were conducted using a Lenovo ThinkPad X390 Yoga (Intel Core i7-8565U CPU @ 1.8 GHz equipped with 16 GB RAM) with Realtek Audio drivers (version 6.0.8757.1). The baseline condition was set by asking participants to complete their tasks without wearing the headphones; in this condition, participants were exposed to the ambient noise of the office space. During this condition, the noise level was measured continuously using a BAFX digital sound meter^31^. The noise meter was positioned immediately behind the participant at ear level. The average ambient noise level across all participants was 42.3 dB with a standard deviation of 1.2 dB.

### Test battery

Five different tests were employed in this study. Cognitive performance assessment included attention using the continuous performance test, learning and inhibition via the Stroop test, and memory using a two-back test. Creativity was evaluated via the remote associate test, and the speed and accuracy of work were measured using a writing performance test. All tests were completed in Psychopy software version 2021.1.0^32^. A brief explanation was added before every test to inform the participant of the task’s nature and to provide brief instructions for proper completion. It is worth noting that the tests did not have a fixed time, because the progress of the test is response-dependent; as soon as the participant provides a response, the next question is presented immediately. A thorough description of each test follows.

#### Continuous performance test

This test measures sustained attention, which is defined as the ability of an individual to focus on a stimulus for a certain period while ignoring distracting stimuli^33^. Previous studies report a moderate reliability level of the continuous performance ranging between 0.4 and 0.7^34^. The test includes 16 different stimuli formed by combinations of four shapes (i.e., star, circle, square, and triangle) and four colors (i.e., yellow, red, white, and blue). During the test, participants were presented with a total of 320 stimuli, each appearing on the screen for 0.3-s followed by a 1-s inter-stimulus (blank screen) period before presenting the next stimulus. Participants were asked to press the “Enter” keystroke whenever they saw the target stimulus: a red star. If a participant failed to react within the 1.3-secs time span when a red star appeared or pressed the “Enter” keystroke when a shape other than the red star was presented, the response was marked as incorrect. The target stimulus accounted for 30% of the images. Color-conjunctive distractors (red non-star) appeared in 17.5% of the trials, and shape-conjunctive (non-red star) appeared in 17.5% of the trials. The remaining 35% of the trials were non-conjunctive distractors, where the shape and color were different from the target stimulus. The order of appearance of stimuli changed every time the test was run to limit any learning effect across the different auditory conditions.

#### Stroop test

This test assesses an individual’s selective attention and inhibition, that is, the capability to overcome a learned response^35^. The Stroop test is widely accepted as a reliable assessment for inhibition and selection attention. For instance, Siegrist^34^ reported a 0.73 reliability for the Stroop test among adults. In this test, participants were presented with 16 different combinations of four-color words in the same four ink colors (i.e., blue, red, yellow, and green). The test consisted of 120 trials, with 50% of the trials showing consistent word and ink color combinations and the remaining 50% presenting color words printed in an inconsistent ink color (e.g., the word “yellow” written in “red” ink). Each word appeared for 1 s followed by 1 s of blank screen before presenting the next word. Participants were required to indicate the ink color, not the color represented by the word itself, with a keystroke of numbers 1 through 4 each associated with a respective color. To help the participants make this association, colored pieces of paper covered each of the number keys according to the color they represent (e.g., a blue piece of paper covered the key for number 1). If a participant failed to react within the 2 s period or pressed the wrong key, the response was marked as incorrect. The order of the trials changed every time the test was run to limit any learning effect across the different auditory conditions.

#### Two-back test

This test assesses working memory^36^. The test is reported to have a moderate to high reliability level^37^. Participants were presented with a sequence of letters and pressed the “Enter” key when the current letter was the same as the letter presented 2 steps earlier in the sequence. The full sequence was composed of 120 letters, each appearing on the screen for 0.5 s followed by a 1.5 s of blank screen before proceeding to the next letter. If a participant failed to react within the 2 s time span when they should have pressed the “Enter” keystroke or pressed the “Enter” keystroke falsely, the response was marked as incorrect. Out of the 120 trials, 30% were target letters while the remaining 70% were non-target. Participants were presented with a different list of letters in each condition to limit any learning effect.

#### Remote associate test

This test assesses creativity levels, particularly an individual’s ability to make associations^38^. The test has been extensively used and is highly reliable. The Spearman-Brown reliability reported by Mednick was 0.92^39^. Participants were presented with three cue words and were asked to determine a fourth word that links the other three words together (e.g., cottage, Swiss and cake are three cues linked by the word “cheese”). Word selections for this study were acquired from a word bank developed from previously published studies^40^. No time limit was allocated for this test; participants could take as much time as they needed to provide an answer before moving to the next set of words. Each trial had only one correct answer, and sores were calculated as the number of correct answers out of 10 trials for each auditory condition.

#### Typing performance test

This test measures an individual’s speed and accuracy in preforming computer work^41^. Participants were presented with a printed paragraph and were asked to type the text into a digital format on a computer. Automatic spelling and grammar checks were disabled in the word processing software during the test. Writing speed was measured as the time needed to type the paragraph, and accuracy was measured as the number of errors made. Participants were provided with a different paragraph of similar size (300 words) and difficulty level (elementary level) for each auditory condition. These paragraphs were acquired from a public resource^42^.

### Electrodermal activity

EDA is a measure of variation in electrical conductance at the surface of the skin^43^. EDA is associated with emotional arousal, stress intensity, and increased cognitive workload of individuals^43^, and therefore, is considered a valid physiological indicator of stress^44^. In this study, EDA was monitored continuously using a wrist band sensor (Empatica E4), which applied an unnoticeable yet continuous voltage to the skin surface to measure variation in skin conductance. EDA was measured in microSiemens (μS) with a sampling frequency of 4 Hz (non-customizable) and a range of 0.01–100 μS^45^. EDA analysis was completed using two components^44^: (1) the tonic component which refers to slow variations in the EDA signal over time measured through the tonic component and (2) the phasic component which refers to rapid and smooth transient events noticeable in the EDA signal. The MATLAB Ledalab toolbox was used to analyze the EDA raw data^46^. The software uses the “Continuous Decomposition Analysis” to decompose the raw EDA data into the tonic and phasic components. The full analysis comprises four steps: estimation of the tonic component, nonnegative deconvolution of phasic SC data, segmentation of driver and remainder, and reconstruction of SC data^47^. However, in this study, the analysis is solely focused on the tonic component of the EDA since the experimental procedure did not include any specific stress-inducing events that required identifying sudden changes via EDA’s phasic component. The tonic component of the EDA data is computed based on the mathematical process of deconvolution^48^, where only data intervals that do not reflect any phasic activity are used to estimate the tonic component. Significant peaks in the EDA data are detected whenever a local maxima shows a difference of 0.2 μS, in comparison to a preceding and succeeding local minima. These peaks are the indicators of phasic activity. Thus, the tonic component is calculated by averaging the values of the driver function governing the EDA data outside the phasic activity intervals. For more details about the “Continuous Decomposition Analysis”, please refer to the following studies: ^47,48^ The difference in the mean tonic activity between baseline EDA and the EDA during the auditory conditions was calculated for every participant. This difference was compared across the different conditions to determine the effect of noise on the EDA.

### Procedure and experimental design

The experiment took place in a private office at a time when no occupants were present in neighboring offices to limit external distractions. The window’s blinds were kept shut and the same artificial lighting conditions were maintained for all participants to limit the effect of lighting on participants’ performance. Similarly, a 24 °C indoor temperature was set and maintained throughout the experiment. This setup mimics a private office setting with standard lighting and thermal conditions and no distracting noise (e.g., no telephone rings, printer noise, chat, etc.) At the outset, participants indicated their gender, age, and dominant hand, reported any sensitivity to noise and completed the online hearing screening. The E4^45^ was placed on the participant’s wrist, and the participant remained still for 5 min to collect an EDA baseline. Then, participants completed the 5 tests in the same order under each auditory condition, starting with the continuous performance test, followed by the Stroop test, two-back test, remote associate test, and finally the typing performance test. Participants were exposed to the 45 dB and 65 dB white noise continuously through the headphones while performing the tests. During the ambient noise condition, participants were asked to complete their tests without wearing the headphones. The study follows a within-subject experimental design, where every participant completed all three conditions. The order of the three auditory conditions was randomized for each participant using a Latin square design^49^. The total duration of the experiment was around 2.5 h.

### Data analysis

A repeated-measures analysis of variance (ANOVA) was used to analyze the outcomes under study during the various auditory conditions as a within subjects’ factor. In this study, the dependent variables are sustained attention, selective attention, inhibition, working memory, creativity, performance, and stress level. The independent variables are the three noise conditions: white noise at 45 dB, white noise at 65 dB, and ambient noise. Tukey HSD analysis was employed to examine the significant differences in the outcomes between each of the three conditions. A p-value of 0.05 was used to determine statistical significance. The statistical analysis was conducted using the IBM SPSS statistics software, version 27^50^.

## Results

### Sustained attention: continuous performance test

Sustained attention was significantly different among the auditory conditions [F(2, 114) = 3.92, *p* = 0.02, d = 0.51, 95% CI [0.01, 0.15]]. Specifically, participants’ scores on the continuous performance test were significantly higher in the 45 dB white noise condition (M = 95.23%, SD = 4.06%) compared to the ambient noise condition (M = 93.12%, SD = 3.34%). No significant differences in sustained attention were found between the 65 dB white noise (M = 93.29%, SD = 3.58%) and the remaining two auditory conditions.

### Selective attention and inhibition: stroop test

No significant effect was noted on participants’ selective attention or inhibition assessed by the Stroop test [F(2, 114) = 0.49 , *p* = 0.61, d = 0.20, 95% CI [0.00, 0.05]].

### Working memory: two-back test

Significant differences in working memory [F(2, 114) = 3.34 , *p* = 0.04, d = 0.46, 95% CI [0.00, 0.14]], were reflected in participants’ scores on the Two-Back test being significantly better in the 65 dB white noise condition (M = 66.38%, SD = 3.74%) compared to the ambient noise condition (M = 64.26%, SD = 3.86%). No significant differences in working memory were found between the 45 dB white noise (M = 64.71%, SD = 3.80%) and the remaining two auditory conditions.

### Creativity: remote associate test

ANOVA results comparing remote associate test scores showed a significant impact of the auditory condition on participants’ creativity levels [F(2, 114) = 3.89 , *p* = 0.02, d = 0.51, 95% CI [0.00, 0.16]]. These differences aligned with sustained attention as being significantly higher with 45 dB white noise (M = 65.13%, SD = 24.69%) compared to ambient noise (M = 50.77%, SD = 24.53%). No significant differences in the creativity measure were found between the 65 dB white noise (M = 54.28%, SD = 27.16%) and the two other auditory conditions.

### Performance: typing performance test

Auditory conditions had a significant effect on both the number of mistakes made by the participants [F(2, 114) = 5.13, *p* = 0.01, d = 0.59, 95% CI [0.01, 0.18]] and the time required to complete the typing task [F(2, 114) = 4.62 , *p* = 0.01, d = 0.55, 95% CI [0.00, 0.17]]. Participants made more mistakes (M = 10.77, SD = 6.21) and took more time (M = 543, SD = 171) when working in the 65 dB white noise condition. This difference was statistically significant compared to the mistakes made during the 45 dB white noise (M = 7.38, SD = 3.92) and ambient noise (M = 7.33, SD = 5.85) and compared to the time required in the 45 dB white noise condition (M = 441, SD = 136). No significant differences were noted between the number of mistakes made under the 45 dB white noise condition and the ambient noise condition, nor in the amount of time required during the ambient noise condition (M = 466, SD = 149) and the two other auditory conditions.

### Stress: change in mean tonic activity

Average changes in the tonic activity from baseline were noted to be different across the conditions [F(2, 114) = 3.26 , *p* = 0.04, d = 0.46, 95% CI [0.00, 0.14]]. Specifically, changes from the baseline tonic activity during the 45 dB white noise condition (M = − 0.20, SD = 0.91), was found to be significantly different than changes in the tonic activity during the 65 dB white noise condition (M = 0.22, SD = 0.74). On the other hand, changes from baseline tonic activity during the ambient noise condition (M = 0.13, SD = 0.62) were not significantly different from the two other auditory conditions.

Average scores for the five tests and changes in the mean tonic activity across the three auditory conditions are provided in Table 1.

**Table 1:** Means, standard deviations, and one-way analyses of variance of the study measures under different noise conditions.

Dependent variable	White noise 45 dB		Ambient noise		White noise 65 dB		*F*(1, 114)	*p* value
	M	SD	M	SD	M	SD		
Sustained attention (% correct)	95.23	4.06	93.12	3.34	93.29	3.58	3.92	0.02
Selective attention and inhibition (% correct)	91.99	8.40	90.11	8.32	90.58	8.64	0.49	0.61
Working memory (% correct)	64.71	3.80	64.26	3.86	66.38	3.74	3.34	0.04
Creativity level (% correct)	65.13	24.69	50.77	24.53	54.28	27.16	3.89	0.02
Performance (number of mistakes)	7.38	3.92	7.33	5.85	10.77	6.21	5.13	0.01
Performance (time in seconds)	441	136	466	149	543	171	4.62	0.01
Δ Mean tonic activity (Microseconds)	− 0.20	0.91	0.13	0.62	0.22	0.74	3.26	0.04

Additionally, a summary of the post hoc analysis is presented in Table 2.

**Table 2:** Post Hoc analysis summary.

Dependent variable	Conditions under comparison	$\left| {{\text{Mean}}\,{\text{difference}}} \right|$	*p* value
Sustained attention (% correct)	WN 45 dB × Ambient noise	0.02	0.03
	WN 45 dB × WN 65 dB	0.01	0.06
	WN 65 dB × Ambient noise	0.01	0.98
Working memory (% correct)	WN 45 dB × Ambient noise	0.01	0.861
	WN 45 dB × WN 65 dB	0.01	0.134
	WN 65 dB × Ambient noise	0.02	0.041
Creativity level (% correct)	WN 45 dB × Ambient noise	0.14	0.038
	WN 45 dB × WN 65 dB	0.14	0.047
	WN 65 dB × Ambient noise	0.01	0.996
Performance (number of mistakes)	WN 45 dB × Ambient noise	0.05	0.999
	WN 45 dB × WN 65 dB	3.42	0.019
	WN 65 dB × Ambient noise	3.43	0.017
Performance (time in seconds)	WN 45 dB × Ambient noise	24.5	0.760
	WN 45 dB × WN 65 dB	101.1	0.012
	WN 65 dB × Ambient noise	76.6	0.074
Δ Mean tonic activity (Microseconds)	WN 45 dB × Ambient noise	0.33	0.147
	WN 45 dB × WN 65 dB	0.42	0.043
	WN 65 dB × Ambient noise	0.09	0.848

## Discussion

This study examined the effect of white noise levels on cognitive performance, creativity, and stress levels in neurotypical young adults, which is not well studied in the literature. In general, white noise level at 45 dB resulted in better cognitive performance in terms of sustained attention, accuracy, and speed of performance as well as enhanced creativity and lower stress levels. The 65 dB white noise condition led only to improved working memory. Results related to creativity, performance and stress levels are especially important to note because white noise condition at 45 dB resulted in significantly better creativity levels compared to the ambient noise at around the same dB level. This points out to the signal characteristics of white noise at 45 dB supporting creativity and shows that white noise condition at 65 dB neither reduces nor improves creativity compared to ambient noise at 45 dB. In addition, white noise condition at 45 dB resulted in significantly better performance both in terms of accuracy and speed compared to the white noise condition at 65 dB. Moreover, participants had lower levels of stress during white noise condition at 45 dB compared to the white noise condition at 65 dB.

Previous studies have shown that white noise results in improved recognition memory^51^, speed of arithmetic computations^52^ and lexical acquisition of novel word forms^9^ in neurotypical adults. Our results extend these findings by showing that white noise at 45 dB and 65 dB enhanced sustained attention and working memory, respectively, in comparison to the ambient noise. Yet, the differences in these two outcomes were not substantial to have major consequences from a practical aspect. Nevertheless, the fact that white noise conditions *–not the ambient noise condition–* triggered better cognitive performance remain worthy of note. This may be as a result of the white noise sound characteristics (pitch and frequency), which makes it resemble the sound of rain, waves or the wind going through tree leaves^53^, and allows it to be perceived as pleasant to the senses^54^ in comparison to the ambient noise. It is worth noting that a general comparison, with the ADHD-related literature, shows that our neurotypical population needed comparatively lower levels of white noise to boost their cognitive performance. For instance, studies examining the effect of white noise on ADHD individuals conducted by Soderlund et al.^55^ and Chen et al.^56^ used 80 dB and 78 dB, respectively, when studying working memory, while Baijot et al.^57^ and Soderlund et al.^58^ used 77 dB and 78 dB, respectively, to study attention. The MBA model postulates that individuals with low dopamine levels require high white noise levels to trigger enhanced internal neural activity but demands less white noise when dopamine activity is high^13^. This explains why researchers focusing on ADHD population (characterized by low dopamine circuity) usually use high white noise levels as well as why enhancements in cognitive functions were noted in our neurotypical population at comparatively lower white noise levels.

Previous studies suggest that low to moderate white noise levels can be enough to induce a high construal level leading to better abstract processing thus enhancing creative thinking^23^. On the other hand, the literature also presents a plethora of studies demonstrating that high levels of white noise can impede the creativity of individuals. For instance, Martindale and Greenough^21^ concluded that a 75 dB white noise resulted in the lowest scores on the remote associate test in comparison to the control (no white noise) condition. Similarly, results from the study conducted by Hillier et al.^20^ showed that a 90 dB white noise would hinder creative thinking compared to the control condition. This is because high white noise levels have been associated with increased distraction, resulting in deteriorated information processing, and thus degraded creativity^23^. In our study, white noise at 65 dB was not too high to impede creativity as suggested by previous studies in related literature. However, our findings show that white noise of 45 dB could support creative thinking in comparison to ambient noise (42.3 dB). This is an important finding as the ambient noise level was relatively equal to the white noise level at 45 dB, which highlights the unique properties of white noise in supporting creativity.

The white noise level at 45 dB allowed for better typing performance in terms of speed and accuracy and led to reduced EDA levels when compared to the white noise at 65 dB. EDA levels have been widely used as indicators of stress in experimental procedures related to environmental interventions^59,60^. Thus, our results support the conclusion that white noise at 45 dB resulted in reduced physiological stress whereas the 65 dB white noise exposure led to increased stress levels while performing cognitively demanding tasks. These findings agree with previous research studies showing that long exposure to high-level white noise results in a stressful response. For example, Liu et al.^61^ showed that the exposure to an 80 dB white noise level was enough to induce more stress than a mental arithmetic task. Similarly, Kraus et al.^62^ argue that at higher noise intensities, activations of sympathetic nervous activity (bodily response to stressors) are prominent. To that end, we postulate that high-stress levels during the 65 dB white noise condition were associated with deteriorated speed and accuracy. This conclusion was further supported by the work of Loewen and Suedfeld^63^ who found that masking office noise with a 60 dB white noise led to high arousal and stress as well as reduced task performance among office workers in comparison to a no-noise condition. On the other hand, our 45 dB white noise condition results are unique; to the best of our knowledge, there has been no study that investigated the effect of low-level white noise on task performance or stress levels among neurotypical adults.

Finally, our results indicate that different tasks might require different white noise levels for optimal performance: at 45 dB white noise level, sustained attention, accuracy, and speed were optimal but working memory improved under the 65 dB white noise level. Research suggests that the necessary dopamine levels for optimal cognitive performance can vary depending on the type of task^9^. For example, memory tasks are usually highly mentally demanding and thus, require higher dopamine levels^64^, which could explain why the 65 dB white noise condition boosted the memory performance of our participants.

While this study contributed to the literature in unique ways as highlighted above, it also holds some limitations. The study did not present enough variability in age and gender to determine the effect of demographics on the relationship between white noise levels and cognitive performance. Also, the study covered two white noise conditions only (45 dB and 65 dB), thus future research directions could perform more studies to uncover the relationship between various white noise levels and cognitive performance, by recruiting more participants as well as examining more white noise levels (e.g., 55 dB and 75 dB) and the personal differences based on gender, age, etc. Moreover, researchers can investigate the effect of different noise colors (e.g., pink, brown, etc.)^65^ on the cognitive performance of neurotypical adults, using the same experimental procedure presented in this paper. Additionally, this study did not perform a concurrent analysis of participants’ dopamine levels to biologically explain the results at hand. To that end, future research efforts can measure dopamine levels to confirm our conclusions and determine the governing relationship between the dopaminergic circuitry and cognitive performance under various white noise levels. This can be accomplished by measuring the concentration of injected radioligand (radioactive biochemical substance) using a positron emission tomography camera which helps detect the dopamine released in the brain^66^. On the practical side, results from this paper can be used to enhance our understanding of customized workspaces. Hence, future research can investigate the means and methods to integrate the use of white noise as a performance booster in the workplace and customize the exposure to different white noise levels to fit into the requirement of the work task at hand.

## Conclusions

This study examined the effect of two white noise conditions, white noise level at 45 dB and white noise level at 65 dB, on the cognitive performance, creativity, and stress levels of neurotypical young adults in a private office space. Our findings showed that white noise level at 45 dB resulted in better cognitive performance in terms of sustained attention, accuracy, and speed of performance as well as enhanced creativity and lower stress levels. On the other hand, the 65 dB white noise condition led to improved working memory but higher stress levels, which leads to the conclusion that different tasks might require different noise levels for optimal performance. These findings are significant, as they extend previous research results about the positive effects white noise has on the cognitive performance of neurotypical adults. Future research directions presented include studying more white noise levels and different noise colors. Similar research might perform a concurrent analysis of participants’ dopamine levels to biologically explain the results.

## Data Availability

The datasets generated and/or analyzed during the current study are not publicly available following the IRB guidelines associated with this study but are available from the corresponding author on reasonable request.

## References

[1] Czanner G (2015). Measuring the signal-to-noise ratio of a neuron. Proc. Natl. Acad. Sci..

[2] Stein RB, Gossen ER, Jones KE (2005). Neuronal variability: Noise or part of the signal?. Nat. Rev. Neurosci..

[3] Baum N, Chaddha J (2021). The impact of auditory white noise on cognitive performance. J. Sci. Med..

[4] Othman E (2019). Low intensity white noise improves performance in auditory working memory task: An fMRI study. Heliyon.

[5] Söderlund, G. Positive effects of noise on cognitive performance: Explaining the moderate brain arousal model. In *The 9th Congress of the International Comisssion on the Biological Effects of Noise* 378–386 (Leibniz Gemeinschaft., 2008).

[6] Sikström S, Söderlund G (2007). Stimulus-dependent dopamine release in attention-deficit/hyperactivity disorder. Psychol. Rev..

[7] Bilder RM, Volavka J, Lachman HM, Grace AA (2004). The catechol-O-methyltransferase polymorphism: Relations to the tonic–phasic dopamine hypothesis and neuropsychiatric phenotypes. Neuropsychopharmacology.

[8] Angwin AJ (2019). White noise facilitates new-word learning from context. Brain Lang..

[9] Angwin AJ (2017). White noise enhances new-word learning in healthy adults. Sci. Rep..

[10] Herweg NA, Bunzeck N (2015). Differential effects of white noise in cognitive and perceptual tasks. Front. Psychol..

[11] Cook A, Bradley-Johnson S, Johnson CM (2014). Effects of white noise on off-task behavior and academic responding for children with ADHD. J. Appl. Behav. Anal..

[12] Söderlund G, Sikström S, Smart A (2007). Listen to the noise: Noise is beneficial for cognitive performance in ADHD. J. Child Psychol. Psychiatry.

[13] Helps SK, Bamford S, Sonuga-Barke EJ, Söderlund GB (2014). Different effects of adding white noise on cognitive performance of sub-, normal and super-attentive school children. PLoS ONE.

[14] U.S. Bureau of Labor Statistics. Occupational Employment and Wages, May 2020. https://www.bls.gov/oes/current/oes430000.htm#nat (2021).

[15] Kidd, A. The Marks are on the Knowledge Worker. In *Proceedings of the SIGCHI Conference on Human Factors in Computing Systems* 186–191 (1994).

[16] Jahncke H, Hallman DM (2020). Objective measures of cognitive performance in activity based workplaces and traditional office types. J. Environ. Psychol..

[17] Fisher GG, Chacon M, Chaffee DS (2019). Theories of Cognitive Aging and Work. Work Across the Lifespan.

[18] Lezak MD, Howieson DB, Loring DW, Fischer JS (2004). Neuropsychological Assessment.

[19] Samani SA, Rasid SZBA, Sofian SB (2015). Individual control over the physical work environment to affect creativity. Ind. Eng. Manag. Syst..

[20] Hillier A, Alexander JK, Beversdorf DQ (2006). The effect of auditory stressors on cognitive flexibility. Neurocase.

[21] Martindale C, Greenough J (1973). The differential effect of increased arousal on creative and intellectual performance. J. Genet. Psychol..

[22] Toplyn G, Maguire W (1991). The differential effect of noise on creative task performance. Creat. Res. J..

[23] Mehta R, Zhu R, Cheema A (2012). Is noise always bad? Exploring the effects of ambient noise on creative cognition. J. Consum. Res..

[24] Nakajima Y, Tanaka N, Mima T, Izumi SI (2016). Stress recovery effects of high-and low-frequency amplified music on heart rate variability. Behav. Neurol..

[25] Reinhardt T, Schmahl C, Wüst S, Bohus M (2012). Salivary cortisol, heart rate, electrodermal activity and subjective stress responses to the Mannheim multicomponent stress test (MMST). Psychiatry Res..

[26] Miyakawa M (2006). Salivary chromogranin A as a measure of stress response to noise. Noise Heal..

[27] Faul F, Erdfelder E, Buchner A, Lang AG (2009). Statistical power analyses using G* power 3.1: Tests for correlation and regression analyses. Behav. Res. Methods.

[28] HearingLife. Online Hearing Test. https://www.hearinglife.com/hearing-loss/your-hearing/test-your-hearing.

[29] Center for Disease Control and Prevention. What Noises Cause Hearing Loss? *Loud Noise Can Cause Hearing Loss* https://www.cdc.gov/nceh/hearing_loss/what_noises_cause_hearing_loss.html#:~:text=Noise above 70 dB over, immediate harm to your ears. (2019).

[30] United States. Office of Noise Abatement Control. *Information on levels of environmental noise requisite to protect public health and welfare with an adequate margin of safety*. (US Government Printing Office, 1974).

[31] Products, B. Advanced: Decibel Meter/Sound Level Reader: W/ Battery! https://bafxpro.com/products/bafx-products-decibel-meter-sound-level-reader-w-battery-advanced-sound-meter.

[32] Peirce JW (2007). PsychoPy: Psychophysics software in Python. J. Neurosci. Methods.

[33] Shalev N, Humphreys G, Demeyere N (2018). Manipulating perceptual parameters in a continuous performance task. Behav. Res. Methods.

[34] Raz S, Bar-Haim Y, Sadeh A, Dan O (2014). Reliability and validity of the online continuous performance test among young adults. Assessment.

[35] Scarpina F, Tagini S (2017). The stroop color and word test. Front. Psychol..

[36] Owen AM, McMillan KM, Laird AR, Bullmore E (2005). N-back working memory paradigm: A meta-analysis of normative functional neuroimaging studies. Hum. Brain Mapp..

[37] Hockey A, Geffen G (2004). The concurrent validity and test? Retest reliability of a visuospatial working memory task. Intelligence.

[38] Mednick MT, Mednick SA, Mednick EV (1964). Incubation of creative performance and specific associative priming. J. Abnorm. Soc. Psychol..

[39] Mednick SA (1968). The remote associates test*. J. Creat. Behav..

[40] Remote Associates Test. *A collection of tasks from the Remote Associates Test of Creativity* https://www.remote-associates-test.com/.

[41] Lan L, Wargocki P, Lian Z (2014). Thermal effects on human performance in office environment measured by integrating task speed and accuracy. Appl. Ergon..

[42] Teachnology, I. Teachnology. https://www.teach-nology.com/.

[43] Prokasy W (2012). Electrodermal Activity in Psychological Research.

[44] Posada-Quintero HF, Chon KH (2020). Innovations in electrodermal activity data collection and signal processing: A systematic review. Sensors.

[45] Empatica. E4 wristband. https://www.empatica.com/research/e4/?gclid=CjwKCAjwtpGGBhBJEiwAyRZX2mR4HYW67O3yXtIcz7eDYJw21RWNNg0pBCJogFM1008kEjhg-3rMvhoCaWoQAvD_BwE (2021).

[46] Benedek M, Kaernbach C (2010). A continuous measure of phasic electrodermal activity. J. Neurosci. Methods.

[47] Benedek M, Kaernbach C (2010). Decomposition of skin conductance data by means of nonnegative deconvolution. Psychophysiology.

[48] Alexander DM (2005). Separating individual skin conductance responses in a short interstimulus-interval paradigm. J. Neurosci. Methods.

[49] Bradley JV (1958). Complete counterbalancing of immediate sequential effects in a latin square design. J. Am. Stat. Assoc..

[50] George D, Mallery P (2019). IBM SPSS Statistics 26 Step by Step.

[51] Rausch VH, Bauch EM, Bunzeck N (2014). White noise improves learning by modulating activity in dopaminergic midbrain regions and right superior temporal sulcus. J. Cogn. Neurosci..

[52] Usher M, Feingold M (2000). Stochastic resonance in the speed of memory retrieval. Biol. Cybern..

[53] Ilkkaya NK (2014). The effects of music, white noise, and ambient noise on sedation and anxiety in patients under spinal anesthesia during surgery. J. Perianesthesia Nurs..

[54] Jastreboff, P. J. Tinnitus habituation Therapy (THI) and Tinnitus Retraining Therapy (THI). In *Tinnitus Handbook* 357–376. (2000).

[55] Söderlund GBW, Björk C, Gustafsson P (2016). Comparing auditory noise treatment with stimulant medication on cognitive task performance in children with attention deficit hyperactivity disorder: Results from a pilot study. Front. Psychol..

[56] Chen I-C (2022). Listening to white noise improved verbal working memory in children with attention-deficit/hyperactivity disorder: A pilot study. Int. J. Environ. Res. Public Health.

[57] Baijot S (2016). Neuropsychological and neurophysiological benefits from white noise in children with and without ADHD. Behav. Brain Funct..

[58] Söderlund GB, Sikström S, Loftesnes JM, Sonuga-Barke EJ (2010). The effects of background white noise on memory performance in inattentive school children. Behav. Brain Funct..

[59] Yin J (2019). Effects of biophilic interventions in office on stress reaction and cognitive function: A randomized crossover study in virtual reality. Indoor Air.

[60] Li D, Sullivan WC (2016). Impact of views to school landscapes on recovery from stress and mental fatigue. Landsc. Urban Plan..

[61] Liu X, Iwanaga K, Shimomura Y, Katsuura T (2007). Comparison of stress responses between mental tasks and white noise exposure. J. Physiol. Anthropol..

[62] Kraus U (2013). Individual daytime noise exposure during routine activities and heart rate variability in adults: A repeated measures study. Environ. Health Perspect..

[63] Loewen LJ, Suedfeld P (1992). Cognitive and arousal effects of masking office noise. Environ. Behav..

[64] Cools R, D’Esposito M (2011). Inverted-U–shaped dopamine actions on human working memory and cognitive control. Biol. Psychiatry.

[65] Azizi A, Yazdi PG, Azizi A, Yazdi PG (2019). Introduction to Noise and its Applications. Computer-Based Analysis of the Stochastic Stability of Mechanical Structures Driven by White and Colored Noise.

[66] Badgaiyan RD (2013). Detection of dopamine neurotransmission in “real time”. Front. Neurosci..

